# Wafer-Level Vacuum-Packaged Electric Field Microsensor: Structure Design, Theoretical Model, Microfabrication, and Characterization

**DOI:** 10.3390/mi13060928

**Published:** 2022-06-11

**Authors:** Jun Liu, Shanhong Xia, Chunrong Peng, Zhengwei Wu, Zhaozhi Chu, Zhouwei Zhang, Hucheng Lei, Fengjie Zheng, Wei Zhang

**Affiliations:** 1 State Key Laboratory of Transducer Technology, Aerospace Information Research Institute, Chinese Academy of Sciences, Beijing 100190, China; liujun173@mails.ucas.ac.cn (J.L.); crpeng@mail.ie.ac.cn (C.P.); zwwu@mail.ie.ac.cn (Z.W.); zhangzhouwei15@mails.ucas.ac.cn (Z.Z.); leihucheng16@mails.ucas.ac.cn (H.L.); fjiezheng@163.com (F.Z.); zhangwei178@mails.ucas.ac.cn (W.Z.); 2 School of Electronic, Electrical and Communication Engineering, University of Chinese Academy of Sciences, Beijing 100049, China; 3 Institute of Microelectronics of Chinese Academy of Sciences, Beijing 100029, China; chuzhaozhi@ime.ac.cn

**Keywords:** wafer-level vacuum packaging, electric field microsensor, MEMS, structural capacitance model

## Abstract

This paper proposes a novel wafer-level vacuum packaged electric field microsensor (EFM) featuring a high quality factor, low driving voltage, low noise, and low power consumption. The silicon-on-insulator (SOI) conductive handle layer was innovatively used as the sensing channel to transmit the external electric field to the surface of the sensitive structure, and the vacuum packaging was realized through anodic bonding between the SOI and glass-on-silicon (GOS). The fabrication process was designed and successfully realized, featured with a simplified process and highly efficient batch manufacturing, and the final chip size was only 5 × 5 mm. A theoretical model for the packaged device was set up. The influence of key parameters in the packaging structure on the output characteristics of the microsensor was analyzed on the basis of the proposed model. Experiments were conducted on the wafer-level vacuum-packaged EFM to characterize its performance. Experimental results show that, under the condition of applying 5 V DC driving voltage, the required AC driving voltage of the sensor was only 0.05 V_P_, and the feedthrough was only 4.2 mV. The quality factor was higher than 5000 and was maintained with no drop in the 50-day test. The vacuum in the chamber of the sensor was about 10 Pa. A sensitivity of 0.16 mV/(kV/m) was achieved within the electrostatic field range of 0–50 kV/m. The linearity of the microsensor was 1.62%, and the uncertainty was 4.42%.

## 1. Introduction

Electric field sensors are widely used in many fields such as meteorology, electric power, aerospace, and petrochemicals [1,2,3,4,5,6], which play an important role in scientific research and production. The microelectromechanical system (MEMS)-based electric field microsensor (EFM) has become one of the hotspots in the research field of electric field sensors [7,8,9,10,11,12,13,14]. Reports on the EFM began in the 1990s, to our knowledge [7]. Since then, researchers have proposed various innovative microsensor structures aiming to improve the sensitivity of the microsensor. For example, Yang proposed the coplanar comb-shaped electrode-resonant EFM with sensitivity of 0.23 mV/kV/m, required driving voltage of 20 V DC and 1 V_P_ (peak voltage) AC, and noise feedthrough of 13.9 mV [11]. Chu proposed an EFM based on torsional resonance with sensitivity of 4.82 mV/kV/m, which is the best level up to now, but the required driving voltage was 30 V DC and 15 V_P_ AC [12]. The microchips were mostly exposed to the external environment for testing and might have been damaged without package protection. The package of the electric field microsensor is also important, but is not fully considered yet.

In recent years, there have been some research reports on EFM’s packaging technology [15,16,17,18]. In 2008, AIST [15] used a metal cap with a hole in the packaging structure of the electric field sensor. The hole on the metal cap allows for the sensing component to be exposed to the external electric field; however, it cannot avoid the influence of external dust and air pressure. In 2012, Zhang [16] proposed a packaging structure for EFM that was composed of a polytetrafluoroethylene (PTEF) cap and a substrate integrated with Kovar alloy. PTEF has good chemical stability and can resist corrosive gases. However, yield and consistency are poor due to the complex back-end fabrication process. In 2021, Wen [17,18] introduced two kinds of microsensor packaging structures aiming to augment the sensor’s sensitivity, but at a much larger, cm size. Research reports are mainly about the chip-level packaging of EFMs, but there are no reports on the wafer-level vacuum packaging (WLVP) of EFMs, which constrains the development of EFM.

WLVP is required to improve manufacturing efficiency and yield, and to reduce driving voltage, the size of the packaged chip for high-density integration, and the cost [19]. WLVP can be realized mainly on the basis of polyimide bonding [20], fusion bonding [21], eutectic bonding [22], metal diffusion bonding [23], and anodic bonding [24]. WLVP is used in some kinds of microsensors, such as pressure sensors, accelerometers, and gyroscopes [25,26,27,28]. For example, Mahmood [27] proposed a wafer-level vacuum-packaged accelerometer based on polyimide bonding, but the usage of polyimide causes charge accumulation, leading to the deviation of results if the design is used for measuring an electric field. Li [28] realized a wafer-level vacuum-packaged pressure microsensor based on bulk micromachining and anodic bonding. However, the process is complex, and a multilayer structure with multiple shapes and various materials changes the electric field distribution, which may cause measurement deviation for EFMs. Thus, the existing designs of WLVP for other microsensors are not directly applicable to EFMs because the electric field distribution on the sensing structure is affected by the packaging structure and materials, and the key technical difficulty in WLVP for EFMs is the construction of the electric field sensing channel with little interference. New methods and novel designs are necessary for the WLVP of EFMs.

This paper proposes a novel wafer-level vacuum-packaged EFM featuring a higher quality factor, lower driving voltage, lower noise, and lower consumption compared with previously reported EFMs [7,8,9,10,11,12,13,14,15,16,17,18]. The silicon-on-insulator (SOI) conductive handle layer was innovatively used as the sensing channel, which can transmit the external electric field to the surface of the sensitive structure without interference. The design of the shielding ring structure and of signal transmission in the GOS substrate can reduce the noise coupled by AC driving voltages. A newly designed fabrication process for EFMs based on SOI and glass-on-silicon (GOS) was also successfully realized, featuring a simplified process and high efficiency in batch manufacturing. Moreover, an improved HF release method for weak stiffness structures is proposed that can be used in the process of a device’s WLVP, and a theoretical structural capacitance model was set up. The microsensor was fabricated, and its experimental characterizations were conducted.

## 2. Structure Design and Working Principle

**Figure 1:**
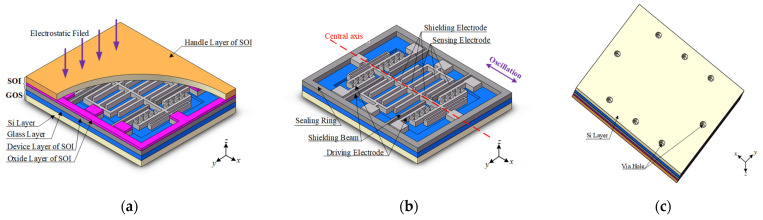
(**a**) Top view of the WLVP structure of the microsensor; (**b**) detailed view of the device layer of SOI; (**c**) bottom view of the WLVP structure of the microsensor.

The schematic view of the proposed WLVP structure is shown in Figure 1. The structure mainly contains three layers: the cap layer, the structure layer, and the substrate layer, as shown in Figure 1a. The cap layer is the conductive handle layer of the SOI, and it is electrically insulated from the structure layer by the buried oxide layer of the SOI. The structure layer is formed by etching the SOI device layer, which is mainly composed of the sealing ring, shielding beams, driving electrodes, and one or more sets of working electrodes, including the sensing electrode and shielding electrode, as shown in Figure 1b. The substrate layer is a GOS composed of a conductive silicon layer and a glass layer. The etched groove on the glass layer provides space for the movement of the shielding electrodes, and via holes are formed in the GOS substrate as shown in Figure 1c.

In the newly developed micromachined wafer-level vacuum-packaged EFM, the SOI conductive handle layer was innovatively used as the sensing channel to transmit the external electric field to the surface of the sensitive structure, and the vacuum packaging was realized through the anodic bonding between SOI and GOS. Moreover, signal transmission was realized in the vertical interconnection hole of the GOS substrate, to be far away from the sealing cap, so as to reduce the interference of the signal to the measured electric field. The shielding ring composed of shielding beams and grounded sealing ring separated the driving and sensing parts, and the silicon layer of the GOS substrate was grounded to reduce the noise coupled by the AC driving power. Through the SOI buried oxygen layer with uniform thickness, the mismatch of the spatial capacitance between the differential driving power and the output is reduced to reduce the crosstalk noise and improve the output stability of the sensor. The wafer-level vacuum packaging structure of the sensor can significantly improve the quality factor of the device, and reduce the driving voltage and noise interference.

**Figure 2:**
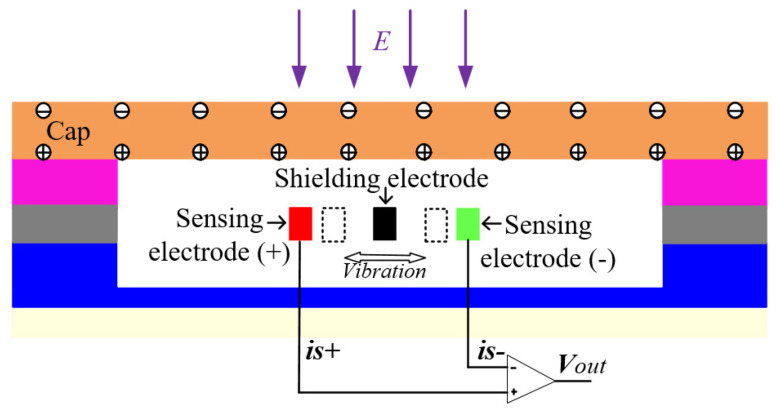
Schematic diagram of the sensor’s working principle.

The working principle of the EFM is based on charge induction. The schematic diagram of the sensor’s working principle is shown in Figure 2. As the cap is a conductive material, a certain amount of induced charge is induced on the outer surface of the cap under the external electric field E. According to the Gaussian theorem, the same amount of heterogeneous charge is induced on the inner surface of the cap at the same time, and the charge on the inner surface of the cap forms an internal electric field in the microchamber [17,29]. When a lateral movement is excited by the push–pull comb drives, the grounded shielding electrodes oscillate back and forth along the central axis, periodically covering the sidewalls of the sensing electrodes to modulate the electric field distribution at the surface of the sensing electrodes. As a result, the quantity of induced charge on the sensing electrodes (+) and the sensing electrodes (−) changes periodically, thereby forming output currents $i_{s}\left(+\right)$ and $i_{s}\left(-\right)$, which are amplified differentially to form output voltage $V_{out}$. Notably, the distortion of the nearby electric field distribution on the sensing electrodes is caused not only by the working electrodes but also by the cap. In other words, the output characteristics of the microsensor are also affected by the packaging structure.

## 3. Theoretical Model

**Figure 3:**
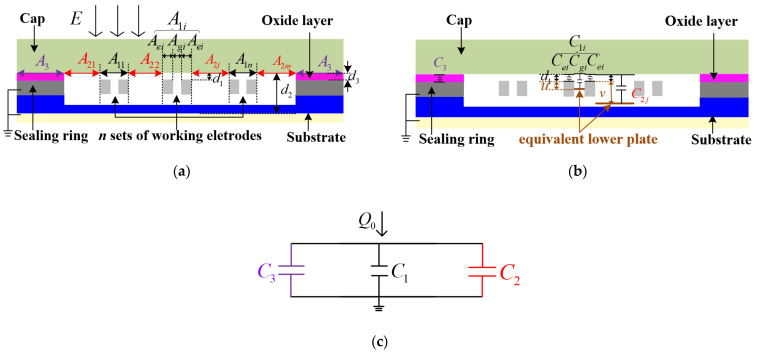
(**a**) Packaging structure model; (**b**) structural capacitors in the packaging structure model; (**c**) capacitance model.

A theoretical model was set up from the perspective of structural capacitance to analyze the output characteristics of wafer-level vacuum packaged EFM. The packaging structure model and the capacitance model are shown in Figure 3.

Figure 3a shows a cross-section of the WLVP structure along the central axis of Figure 1a. In Figure 3a, $A_{1}$ represents a part of the cap’s inner surface area, directly below which were the working electrodes, and $A_{1}=\overset{n}{\underset{i=1}{\sum }}A_{1i}$ for n sets of working electrodes. $d_{1}$ is the gap between the cap and the working electrodes. $A_{1i}$ consisted of three parts and $A_{1i}=2A_{ei}+A_{gi}$. Thus, $A_{1}$ can be given by

(1) $$A_{1}=\overset{n}{\underset{i=1}{\sum }}A_{1i}=\overset{n}{\underset{i=1}{\sum }}\left(2A_{ei}+A_{gi}\right)$$

$A_{2}$ represents a part of the cap’s inner surface area, below which is the gap between two adjacent sets of working electrodes or the gap between the working electrodes and the sealing ring, and $A_{2}=\overset{m}{\underset{j=1}{\sum }}A_{2j}$. $d_{2}$ is the gap between the cap and the GOS substrate’s silicon layer.

$A_{3}$ represents a part of the cap’s inner surface area, directly below which is the sealing ring. $d_{3}$ is the gap between the cap and the sealing ring and $d_{3}=d_{1}$.

Figure 3b shows the structural capacitors in the packaging structure. $C_{1i}$ represents the capacitance of the capacitor between area $A_{1i}$ of the cap and the working electrodes. As shown in Figure 3b, $C_{1i}$ consisted of three parts and $C_{1i}=2C_{ei}+C_{gi}.$ $C_{ei}$ represents the capacitance of a parallel plate capacitor between area $A_{ei}$ and the upper surface of working electrodes, so $C_{ei}=\frac{\varepsilon_{0}A_{ei}}{d_{1}}$ [30]. $C_{gi}$ represents the capacitance of the capacitor between area $A_{gi}$ and sidewalls of the working electrodes. For simplicity, we calculated $C_{gi}$ as an equivalent parallel plate capacitor and an equivalent lower plate is introduced to simulate the lower plate of $C_{gi}$. The gap between the cap and the equivalent lower plate of $C_{gi}$ was $d_{1}+u$, and $C_{gi}$ could be calculated as $C_{gi}=\frac{\varepsilon_{0}A_{gi}}{d_{1}+u}$. Thus, $C_{1}=\overset{n}{\underset{i=1}{\sum }}C_{1i}$ for n sets of working electrodes can be expressed as

(2) $$C_{1}=\overset{n}{\underset{i=1}{\sum }}C_{1i}=\overset{n}{\underset{i=1}{\sum }}\left(2C_{ei}+C_{gi}\right)=\overset{n}{\underset{i=1}{\sum }}\left(2\frac{\varepsilon_{0}A_{ei}}{d_{1}}+\frac{\varepsilon_{0}A_{gi}}{d_{1}+u}\right)$$

$C_{2j}$ represents the capacitance of the capacitor between area $A_{2j}$ of the cap and the substrate. $C_{2j}$ cannot be calculated as $C_{2j}=\frac{\varepsilon_{0}A_{2j}}{d_{2}}$ [30] considering the capacitors between area $A_{2j}$ and sidewalls of the working electrodes. For simplicity, we calculated $C_{2j}$ as an equivalent parallel plate capacitor, and an equivalent lower plate was introduced to simulate the lower plate of $C_{2j}$. The gap between the cap and the equivalent lower plate of $C_{2j}$ is $d_{1}+v$ and $C_{2j}$ can be calculated as $C_{2j}=\frac{\varepsilon_{0}A_{2j}}{d_{1}+v}$. Thus, $C_{2}=\overset{m}{\underset{j=1}{\sum }}C_{2j}$ can be expressed as

(3) $$C_{2}=\overset{m}{\underset{j=1}{\sum }}C_{2j}=\overset{m}{\underset{j=1}{\sum }}\frac{\varepsilon_{0}A_{2j}}{d_{1}+v}$$

$C_{3}$ represents the capacitance of the capacitor between the cap and the seal ring and $C_{3}=\frac{2\varepsilon_{0}\varepsilon_{r}A_{3}}{d_{3}}$ [30].

Figure 3c shows the capacitance model inferred from Figure 3b. $Q_{0}=Q_{1}+Q_{2}+Q_{3}=\varepsilon_{0}EA=\varepsilon_{0}E\left(A_{1}+A_{2}+2A_{3}\right)$ [30], where E is the external electric field, A is the total area of the cap’s inner surface, $Q_{0}$ is the total quantity of charge on the cap’s inner surface, $Q_{1}$ is the quantity of charge in area $A_{1}$, $Q_{2}$ is the quantity of charge in area $A_{2}$, $Q_{3}$ is the quantity of charge in area $2A_{3}$. In order to obtain the charge distribution on the inner surface of the cap, we calculated $Q_{1}$ and $Q_{2}$ according to the capacitance model in Figure 3c, which can be expressed as

(4) $$Q_{1}=Q_{0}\frac{C_{1}}{C_{1}+C_{2}+C_{3}}=\varepsilon_{0}EA\frac{\frac{2A_{e}}{d_{1}}+\frac{A_{g}}{d_{1}+u}}{\frac{2\varepsilon_{r}A_{3}+2A_{e}}{d_{1}}+\frac{A_{2}}{d_{1}+v}+\frac{A_{g}}{d_{1}+u}}$$

(5) $$Q_{2}=Q_{0}\frac{C_{2}}{C_{1}+C_{2}+C_{3}}=\varepsilon_{0}EA\frac{\frac{A_{2}}{d_{1}+v}}{\frac{2\varepsilon_{r}A_{3}+2A_{e}}{d_{1}}+\frac{A_{2}}{d_{1}+v}+\frac{A_{g}}{d_{1}+u}}$$

where $A_{e}=\overset{n}{\underset{i=1}{\sum }}A_{ei}$, $A_{g}=\overset{n}{\underset{i=1}{\sum }}A_{gi}$.

When the shielding electrodes move along the central axis by a distance of L away from the sensing electrodes, $Q_{1}$ increases and $Q_{2}$ decreases, $Q_{1}$ turns into $Q_{1}{}^{\prime }$, and $Q_{2}$ turns into $Q_{2}{}^{\prime }$. In other words, the distance between the shielding electrode and the sensing electrode was minimal in the initial state, and the distance became maximal after the offset of L. Notably, L is the maximal offset of the shielding electrodes. $Q_{1}{}^{\prime }$ and $Q_{2}{}^{\prime }$ can be given by

(6) $$Q_{1}{}^{\prime }=\varepsilon_{0}EA\frac{\frac{2A_{e}}{d_{1}}+\frac{A_{g}+L}{d_{1}+u}}{\frac{2\varepsilon_{r}A_{3}+2A_{e}}{d_{1}}+\frac{A_{2}-L}{d_{1}+v}+\frac{A_{g}+L}{d_{1}+u}}$$

(7) $$Q_{2}{}^{\prime }=\varepsilon_{0}EA\frac{\frac{A_{2}-L}{d_{1}+v}}{\frac{2\varepsilon_{r}A_{3}+2A_{e}}{d_{1}}+\frac{A_{2}-L}{d_{1}+v}+\frac{A_{g}+L}{d_{1}+u}}$$

The charge in area $A_{1}$ was almost coupled to the working electrodes, and the charge in area $A_{2}$ was coupled to the working electrodes and the substrate. Therefore, the quantity of charge at the surface of the working electrodes could be approximated as $Q_{1}+\left(Q_{2}-Q_{2substrate}\right)$ according to the Gaussian theorem, where $Q_{2substrate}$ is the quantity of the charge coupled to the substrate, which can nearly seem to be a constant when the shielding electrodes move. Therefore, the peak of induced charge variation on the surface of the working electrodes was $\Delta Q=\Delta Q_{1}-\left(\Delta Q_{2}-\Delta Q_{2substrate}\right)\approx \Delta Q_{1}-\Delta Q_{2}=\left(Q_{1}{}^{\prime }-Q_{1}\right)-\left(Q_{2}-Q_{2}{}^{\prime }\right)$. $\Delta Q_{1}$ and $\Delta Q_{2}$ can be given by

(8) $$\Delta Q_{1}=\varepsilon_{0}EA\left(\frac{w+L}{\left(z+\frac{v-u}{d_{1}+v}L\right)}-\frac{w}{z}\right)=\varepsilon_{0}EA\frac{L\left(z-w\frac{v-u}{d_{1}+v}\right)}{z\left(z+\frac{v-u}{d_{1}+v}x\right)}$$

(9) $$\Delta Q_{2}=\varepsilon_{0}EA\frac{\frac{d_{1}+u}{d_{1}+v}A_{2}}{z}-\varepsilon_{0}EA\frac{\frac{d_{1}+u}{d_{1}+v}\left(A_{2}-L\right)}{\left(z+\frac{v-u}{d_{1}+v}L\right)}$$

where $w=2A_{e}\frac{d_{1}+u}{d_{1}}+A_{g},\;z=2\varepsilon_{r}A_{3}+A_{1}+A_{2}+\frac{u}{d_{1}}(2\varepsilon_{r}A_{3}+2A_{e})-\frac{v-u}{d_{1}+v}A_{2}$.

When the shielding electrodes of the microsensor are harmonically actuated, the induced charge on the sensing electrodes can be expressed as *Q*(*t*) = *Q*_0_ + *Q*_*A*_ sin (*wt*), where $Q_{0}$ is the initial charge on the sensing electrodes, and $Q_{A}$ is the amplitude of the charge variation on the sensing electrodes [11]. In this paper, $Q_{A}=\frac{1}{2}\times \frac{\Delta Q}{2}=\frac{\Delta Q_{1}-\Delta Q_{2}}{4}$, so the output current of the microsensor can be expressed as [31]

$$i=\frac{d\left(Q\left(t\right)\right)}{dt}=w\frac{\Delta Q_{1}-\Delta Q_{2}}{4}\cos\left(wt\right)$$

(10) $$=\frac{w}{4}\varepsilon_{0}AL\frac{v-u}{d_{1}+v}\frac{L\left(z-w-\frac{d_{1}+u}{d_{1}+v}A_{2}\right)}{z\left(z+\frac{v-u}{d_{1}+v}L\right)}E\cos\left(wt\right)\;$$

## 4. Analysis of Structural Parameters and Simulation

Equation (10) shows that the output current of the microsensor was proportional to the peak of the charge variation of the working electrodes, which is $\Delta Q=\Delta Q_{1}-\Delta Q_{2}$, so we could optimize the packaging structure through the comparison of ΔQ under different structural dimensions. The main key parameters in the packaging structure are $d_{1}$ and $A_{3}$. In this section, we analyze and optimize the key structural parameters on the basis of both the structural capacitance theoretical model proposed in the above section and the finite-element simulation.

### 4.1. Gap between Cap and Working Electrodes

**Figure 4:**
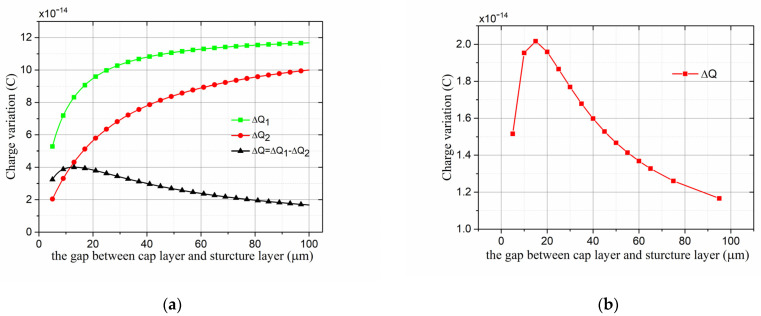
(**a**) Theoretical analysis results of $\Delta Q_{1}$, $\Delta Q_{2}$ and $\Delta Q=\Delta Q_{1}-\Delta Q_{2}$ versus the gap ($d_{1}$) between the cap and working electrodes. $\Delta Q_{1}$ and $\Delta Q_{2}$ are defined in Equations (8) and (9). (**b**) Simulation result of $\Delta Q=\Delta Q_{1}-\Delta Q_{2}$ versus the gap ($d_{1}$) between the cap and working electrodes.

In the theoretical model mentioned above, we set the gap ($d_{1}$) between the cap and the working electrodes as the variable, and other parameters as constants in Equations (8) and (9) to theoretically analyze the influence of $d_{1}$ on $\Delta Q=\Delta Q_{1}-\Delta Q_{2}$. Theoretical analysis results of $\Delta Q_{1}$, $\Delta Q_{2}$, and ΔQ versus $d_{1}$ are shown in Figure 4a. Results were similar when u and v take any value within a reasonable range. Results indicate that $\Delta Q_{1}$ and $\Delta Q_{2}$ increased as $d_{1}$ increased, but $\Delta Q=\Delta Q_{1}-\Delta Q_{2}$ increased first and then decreased as $d_{1}$ increased, and ΔQ was maximized when $d_{1}$ was in the range of 10 to 20 µm.

The finite-element simulation was conducted in ANSYS 18.2 software on the basis of the two-dimensional (2D) simulation model, constructed according to the packaging structure in Figure 3a. The size parameters in the model were set according to actual device’s size parameters shown in Table 1, and the material properties of each part of the structure were set to be the same as those of the actual device. In essence, the shielding electrodes modulate the electric field distribution on the sensing electrodes by lateral displacement. Therefore, the simplified 2D simulation model was a typical cross-section of the three-dimensional (3D) microsensor, and the rules of the electric field distribution in the 2D simulation model could represent that in the whole 3D microsensor. In the simulation process, the 2D geometric model was constructed in ANSYS. The upper boundary of the air case was loaded with voltage, the lower boundary of the air case was grounded to form an electric field of 1 kV/m, and the left and right boundaries of the air case were set at infinity to form a uniform electric field. The cap, buried oxide, and GOS glass layer were float potential, and the working electrodes, sealing ring, and GOS silicon layer were grounded in the simulation model. The meshing size was set to be 1 µm around the sensing electrodes for more accurate results, and the electrical analysis module of ANSYS was used. In the finite-element simulation analysis of $d_{1}$, we set other parameters as constants, and changed the value of $d_{1}$. The simulation result of ΔQ versus $d_{1}$ is shown in Figure 4b. The result indicates that ΔQ first increased and then decreased as $d_{1}$ increased. The simulation result agrees with the theoretical analysis result.

Although a larger output current was achieved when $d_{1}$ was in the range of 10 to 20 µm, it was hard to change the value of $d_{1}$ because it was the thickness of the SOI buried oxide layer. An SOI with a 2 µm thick buried oxide layer was used in this paper for device manufacturing due to limited laboratory conditions. An SOI with a thicker buried oxide layer can be used to improve the performance of the microsensor.

### 4.2. Width of the Sealing Ring

**Figure 5:**
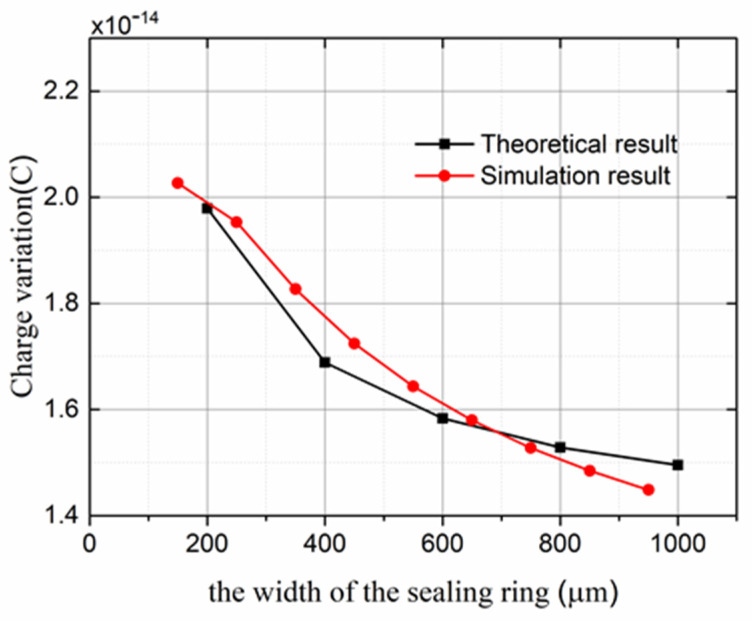
Theoretical-analysis and simulation results of $A_{2j}$ versus the width of the sealing ring ($A_{3}$), $A_{2j}$ is the peak of induced charge variation on the surface of the working electrodes.

In order to study ΔQ versus the width of the sealing ring ($A_{3}$), in both theoretical analysis and the finite-element simulation, we set $A_{3}$ to be the variable, and other parameters to be constants to analyze the influence of $A_{3}$ on ΔQ. The simulation conditions were the same as those in Section 4.1. The theoretical-analysis and finite-element simulation results of ΔQ versus $A_{3}$ are shown in Figure 5. Results indicate that ΔQ decreased as $A_{3}$ increased, and the simulation result agrees with the theoretical analysis result. In order to increase the output current of the microsensor, it is necessary to reduce the width of the sealing ring as much as possible, but a reduction in sealing ring size may result in a decline in packaging quality. The width of the sealing ring was lastly chosen to be 250 µm after many experiments.

For the other parameters of $A_{1}$, $A_{2}$, and so on, the theoretical and simulation models could be also set up to explain some conclusions that can only be explained by simulations or not revealed before. The key parameters of the microsensor are shown in Table 1.

### 4.3. Resonant-Mode Simulation

**Figure 6:**
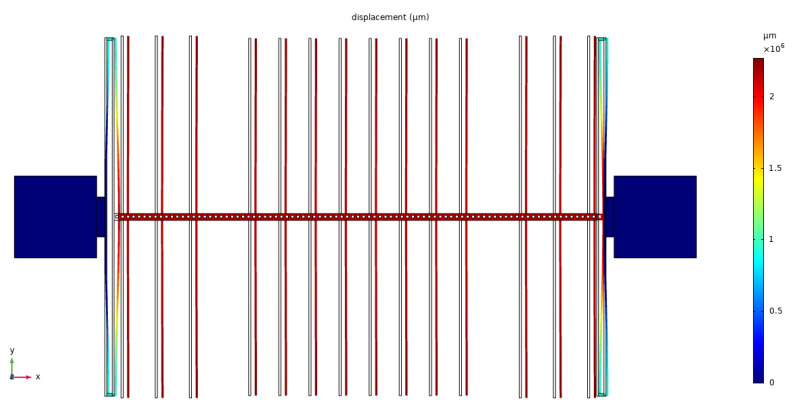
First-order resonant mode of the microsensor by simulation. Displacement is magnified for clear display in COMSOL.

In order to obtain large vibration, the microsensor was designed to work at the resonant frequency. In COMSOL 5.4 software, the 3D simulation model was constructed according to the structure in Figure 3a. The size parameters in the model were set according to the actual device’s size parameters shown in Table 1, and the material properties of each part structure were set to be the same as those of the actual device. The anchor was fixed, the resonant structure was set to free, the meshing size was set to be 3.74 µm in the minimal unit and 137 µin the maximal unit, and the modal analysis module in COMSOL was used. The frequencies of the first six orders were achieved by simulation, which were 5227.9, 12,190, 15,442, 17,342, 21,517, and 23,641 Hz. In Figure 6, the colors represent the displacement magnitudes of the resonant structure. The dark blue part is the initial position without displacement, and the colored part is the position after vibration. The mode shown in Figure 6 was the first because its resonant frequency of 5227.9 Hz was the lowest, which could be directly obtained with the modal analysis module in COMSOL. The first mode was the lateral vibration mode, which is required according to the working principle of the sensor and was chosen to be the mode of operation. The resonant frequency of the lateral vibration mode was 5227.9 Hz, which is far from the second-order resonant frequency of 12,190 Hz. Therefore, the other resonant modes were hardly excited during the microsensor’s operation, which reduced the interference between the working mode and the other modes.

## 5. Device Fabrication

**Figure 7:**
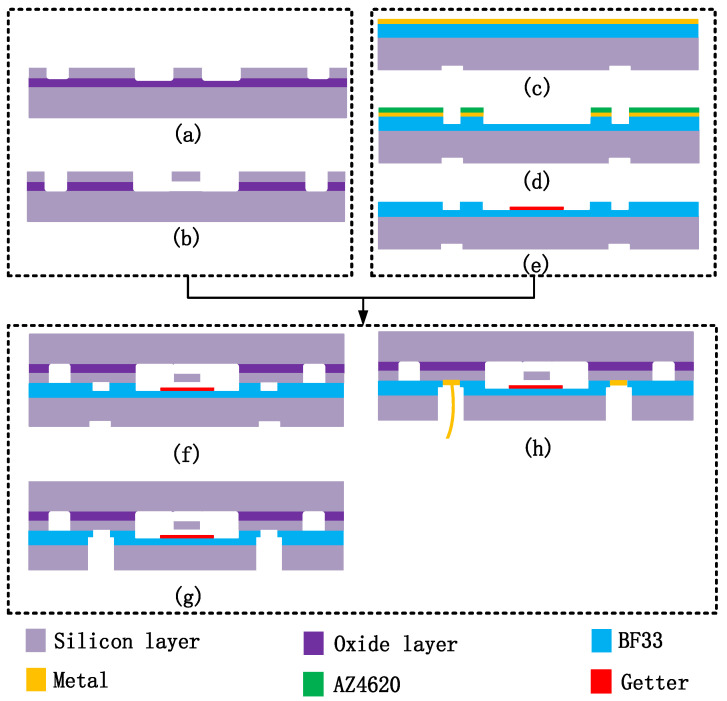
Main steps of the microfabrication process.

We completed the microfabrication process of the wafer-level vacuum-packaged EFM in our laboratory by breaking through some key processes. The fabrication of the sensitive structure and the vacuum packaging of the microsensor were realized by batch manufacturing. The microfabrication process was mainly based on GOS, SOI, and anodic bonding technologies. The GOS was anodically bonded by a 300 µm thick silicon wafer and a 300 µm thick BF33 glass wafer, and the glass layer of the GOS was then thinned to 100 µm by chemical mechanical polishing (CMP). The main steps of fabrication shown in Figure 7 are described as follows (and the details can be seen in the Supplementary Material):

(a) Deep reactive ion etching (DRIE) was utilized to etch the SOI device layer to form the sealing ring, driving electrodes, shielding beams, and working electrodes.

(b) The oxide layer below the driving electrodes and the working electrodes was removed by vapor HF.

(c) Cr/Au with thickness of 30/200 nm was deposited on the glass surface of the prepared GOS wafer to form the protective layer. Then, reactive-ion etching (RIE) with SF_6_ gas was used to form alignment marks on the silicon surface of the GOS wafer, which was used for the alignment of anodic bonding.

(d) The Cr/Au deposited on the glass surface of the GOS was patterned using the metal corrosive fluid with the protection of the patterned AZ4620 photoresist to form the mask. Then, the glass layer of the GOS was patterned utilizing vapor HF with the protection of the patterned Cr/Au mask.

(e) Ti/Au with thickness of 1 µm/30 nm getter was deposited with the protection of a hard mark, which was patterned by a laser process.

(f) The anodic bonding of the SOI and GOS wafers in a vacuum environment was conducted, and the Ti/Au getter was activated during the bonding process. The bonding voltage was 400 V, the bonding temperature was 350 °C, and the bonding pressure was 500 mbar.

(g) DRIE was utilized to etch completely through the GOS’s silicon layer from the backside, and the etch stopped at the surface of the GOS’s glass layer. Then, vapor HF was utilized to etch the exposed GOS’s glass layer from the backside with the protection of the patterned GOS’s silicon layer.

(h) Al metal pads with thickness of 1 µm were deposited in the via holes with the protection of another hard mark, and wire bonding was conducted.

In Step (b), due to the weak stiffness of the structure, the adhesion of the beams appeared while utilizing the two-step process of HF etching and water removal with isopropyl alcohol (IPA) [32]. Therefore, we propose an improved HF release process: after several cycles of the traditional two-step process, isopropyl alcohol is no longer used, but the hot-plate heating method is adopted to remove water vapor, so as to prevent the combination of volatile IPA and water vapor, which causes the adhesion of the beams. The improved method can be used for the release of other weak-stiffness structures.

**Figure 8:**
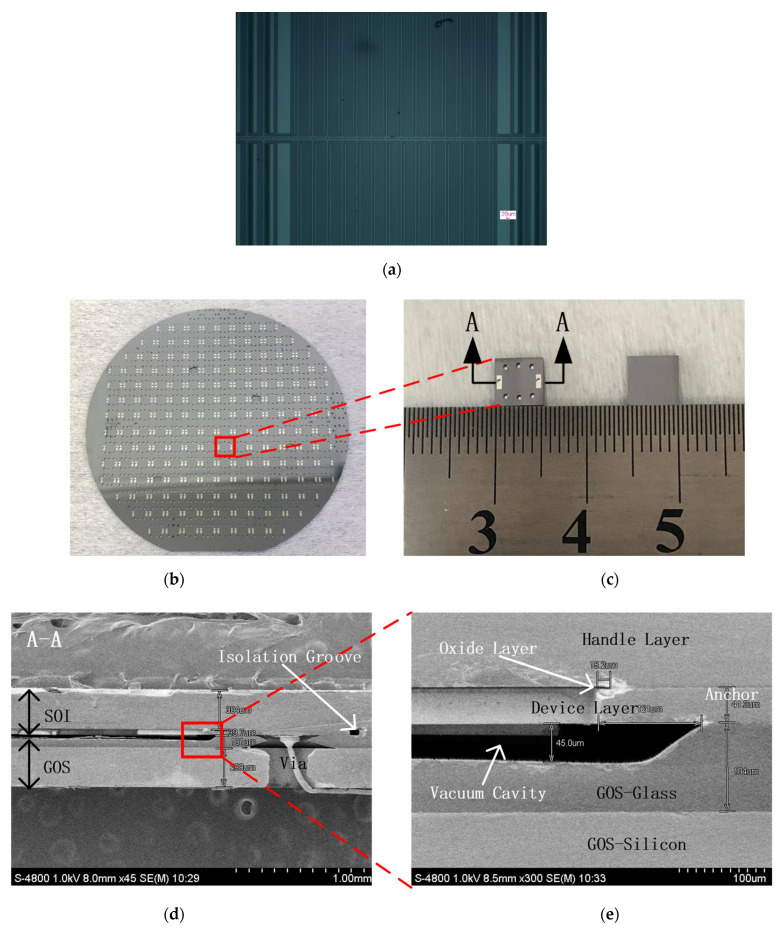
(**a**) Sensitive structure of EFM; (**b**) bonded SOI and GOS wafer; (**c**) back and front views of the chip after dicing; (**d**,**e**) cross-sectional SEM photographs of the bonded SOI and GOS wafer.

Figure 8a shows the sensitive structure of EFM after the removal of the buried oxide. Figure 8b shows the bonded SOI and GOS wafer. Figure 8c shows the back and front views of the chip after dicing. Figure 8d,e show cross-sectional scanning electron micrograph (SEM) photographs of the bonded SOI and GOS wafer. The chip size was 5 × 5 mm. The thickness of each layer was consistent with the design considering the fabrication process error. The yield was higher than 80%, which showed the high efficiency of the batch manufacturing.

## 6. Experimental Results

### 6.1. Experimental Setup

**Figure 9:**
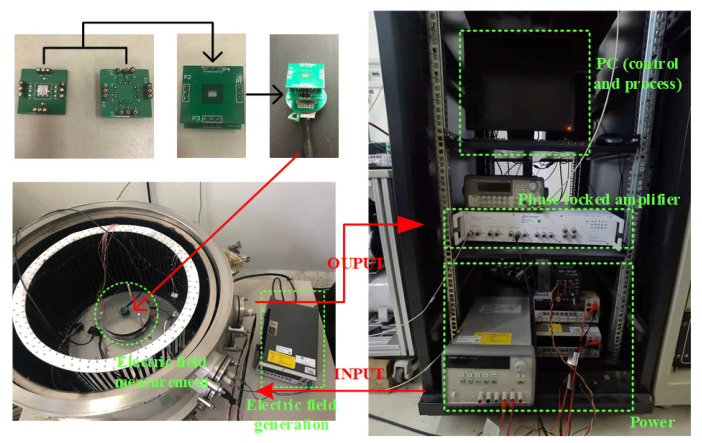
Photographs of the test system.

As shown in Figure 9, the test system was composed of a PC, phase-locked amplifier, power section, electric field generation section, and electric field measurement section. The power section provides power to the interface circuits, and DC driving signal to the microsensor. The AC driving signal is provided by the phase-locked amplifier. In addition, the standard uniform electrostatic field is generated according to IEEE Std 1227-1990 by applying voltage between two parallel metal plates using a signal generator.

**Figure 10:**
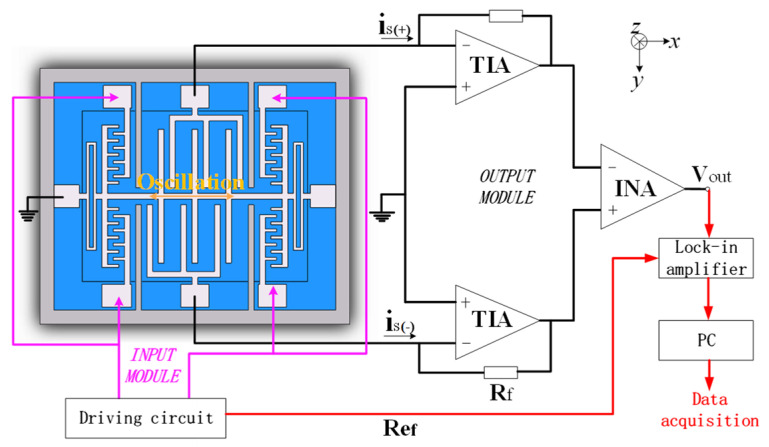
Detection schematic. TIA, transimpedance amplifier; INA, instrumentation amplifier.

The detection schematic is shown in Figure 10. The output current signal of the microsensor is I/V converted and differential amplified through the interface circuits, and then transmitted into the HF2LI dual-channel lock-in amplifier produced by Zurich Instruments. Lastly, the signal is processed in the phase-locked amplifier and then transmitted to the PC.

### 6.2. Characterization Results

**Figure 11:**
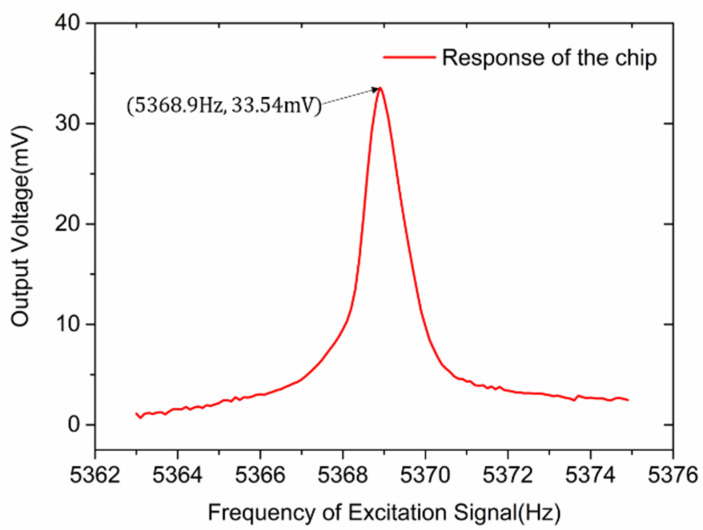
Amplitude–frequency response of the microsensor.

The tested amplitude–frequency response of the microsensor is shown in Figure 11, and the tested resonant frequency was 5368.9 Hz. The frequency of the lateral vibration mode was 5227.9 Hz by simulation. The test result of resonant frequency was in good accordance with the simulation result. The discrepancy is related to the fabrication process variation and the simulation deviation.

The quality factor of the vacuum-packaged EFM proposed in this paper was calculated to be 5738, while the quality factor of the unpackaged EFM was approximately 60 at atmospheric pressure [10]. We tested the unpackaged EFM in a chamber in which the pressure had been controlled. When the pressure in the chamber was about 10 Pa, the Q value of the unpackaged EFM was close to the Q value of the wafer-level vacuum-packaged EFM. Therefore, it could be inferred that the vacuum degree inside the wafer-level vacuum-packaged EFM was about 10 Pa.

Experimental results show that driving signals with 5 V DC voltage and 0.05 V_P_ AC voltage are required for the microsensor proposed in this paper, while driving signals with 20 V DC voltage and 1 V_P_ AC voltage are required for the unpackaged EFM [11]. The feedthrough signal at the output of the microsensor in this paper was 4.2 mV, which was lower than the feedthrough signal of 13.9 mV in the previously reported unpackaged EFM [11]. The vacuum packaging can reduce the damping, so only a 0.05 V_P_ AC driving signal is required, and noise reduction designs are carried out in the structure. Therefore, the noise coupled by the AC driving signal is greatly reduced. The noise is not further reduced due to the noise in the circuit itself, which can be improved with the optimization of the circuit. Sensor consumption was in the order of mW or lower due to the decrease in driving voltages.

**Figure 12:**
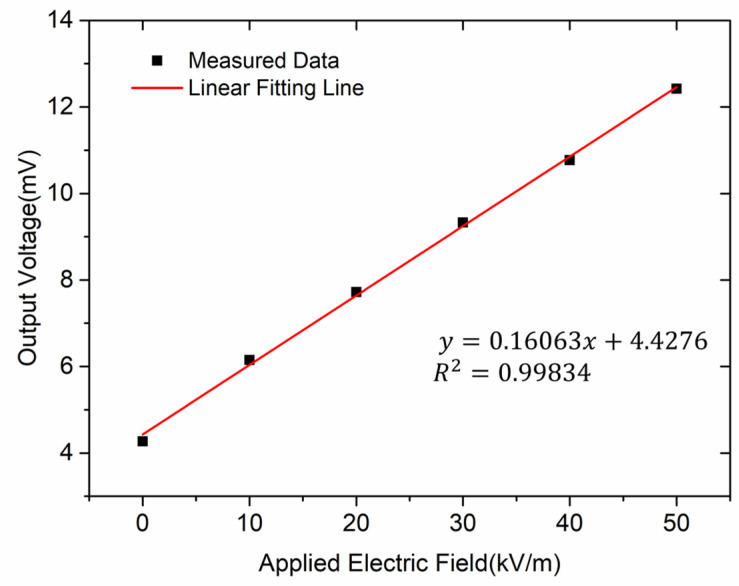
Response of the microsensor versus the applied electric field.

Figure 12 shows the response of the microsensor versus the applied electric field when the microsensor works in resonant mode. The experimental result shows that the measuring range of the microsensor was 0–50 kV/m, and the sensitivity of the microsensor was 0.16 mV/(kV/m).

**Figure 13:**
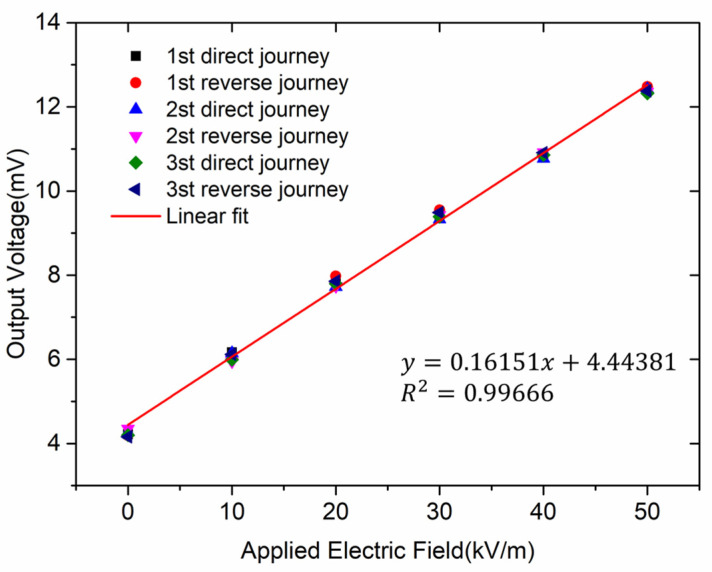
Response of the microsensor versus the applied electric field in three roundtrip measurements.

Furthermore, the microsensor was tested in three roundtrip measurements, and results are shown in Figure 13. A roundtrip is composed of a direct journey and a reverse journey. The electric field was applied from 0 to 50 kV/m in the direct journey, and from 50 kV/m to 0 in the reverse journey. The microsensor demonstrated good performance with a linearity of 1.62% and an uncertainty of 4.42%.

**Figure 14:**
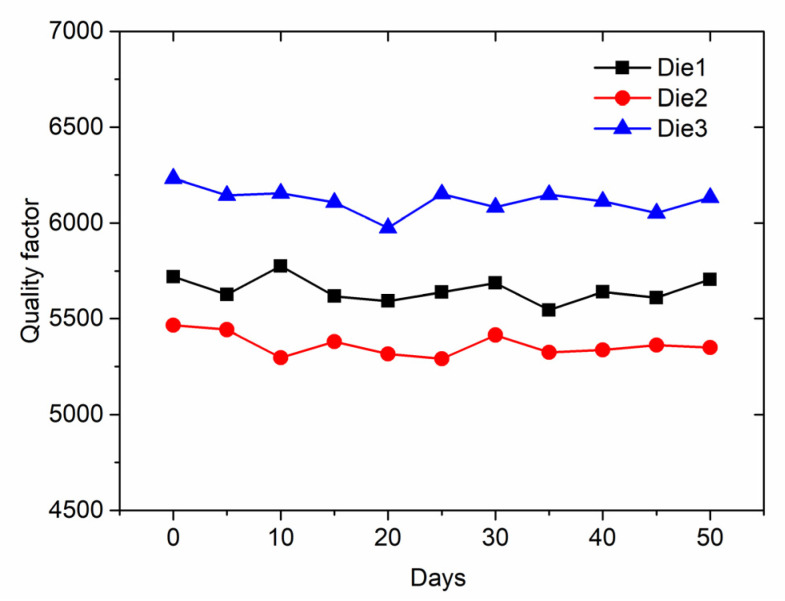
Quality factors of three microsensors (Dies 1–3) versus time.

Three dies were selected, and quality factors were measured every five days during the 50-day test. Quality factors of the microsensors versus time are shown in Figure 14. Results show that the quality factors had no obvious drop and were kept in a certain range during the 50-day test. The test result indicates that reliable packaging without leakage was achieved.

The comparison between previously reported EFMs and this work is shown in Table 2. The feedthrough reported by Yang was previously the best level, to our knowledge. The previously reported EFMs all work at atmospheric pressure, and quality factors are low. Therefore, driving signals were reduced in this work. The quality factor was improved, and the feedthrough was reduced in this work.

## 7. Conclusions

In this paper, a novel wafer-level vacuum-packaged electric field microsensor was proposed and successfully fabricated. The sensor requires low driving voltages to reduce power consumption, and achieved higher quality factor and lower noise feedthrough compared with previously reported EFM. The SOI conductive handle layer was innovatively used as the sensing channel to reduce interference. The newly designed fabrication process based on SOI and GOS was successfully realized, featuring a simplified process and high efficiency in batch manufacturing, which could be used for reference for other kinds of WLVP microsensors. The proposed improved HF release method can be used for the high-quality release of other weak-stiffness structures. A theoretical structural capacitance model was set up to analyze the sensitive mechanism and characteristics of the microsensor, which are of great significance to study the influence of the package structure on EFMs.

Experimental results show that the final chip size was only 5 × 5 mm; under the condition of applying 5 V DC driving voltage, the required AC driving voltage of the sensor was only 0.05 V_P_, and the feedthrough was only 4.2 mV. The quality factor was higher than 5000 and was maintained with no drop in the 50-day test.

The proposed microsensor could be used in high-density integrated devices due to its small size, such as by being integrated into a mobile phone. The proposed microsensor can also be used in some practical application scenarios that require long-term use and long-term stability, such as power transmission and meteorological sounding, due to its low driving voltages, low power consumption, and low noise. In the future, we will further improve the sensor’s sensitivity by optimizing the packaging structure, and further carry out application verification for practical application scenarios.

## Figures and Tables

**Table 1:** Key parameters of the proposed EFM.

Structural Parameters	Value
$\mathrm{Distance}\;\mathrm{between}\;\mathrm{cap}\;\mathrm{and}\;\mathrm{working}\;\mathrm{electrodes}\;d_{1}$	2 µm
$\mathrm{Width}\;\mathrm{of}\;\mathrm{sealing}\;\mathrm{ring}\;A_{3}$	250 µm
Thickness of the structure layer	40 µm
$\mathrm{Width}\;\mathrm{of}\;\mathrm{working}\;\mathrm{electrodes}\;A_{ei}$	9 µm
Length of the working electrodes	635 µm
Gap between shielding electrode and $\mathrm{sensing}\;\mathrm{electrode}\;A_{gi}$	15 µm
$\mathrm{Gap}\;\mathrm{between}\;\mathrm{two}\;\mathrm{adjacent}\;\mathrm{sets}\;\mathrm{of}\;\mathrm{working}\;\mathrm{electrodes}\;A_{2j}$	75 µm
Number of working electrodes	8 × 2
Width of driving electrodes	9 µm
Length of driving electrodes	635 µm
Number of driving electrodes	6 × 2

**Table 2:** Comparison with previously reported EFM.

Reported EFM	Year	Driving Signals	Quality Factor	Feedthrough
Riehl [8]	2003	DC 30 V and AC 2.5 V_P_	-	19.1 mV
Yang [11]	2011	DC 20 V and AC 1 V_P_	60	13.9 mV
Chu [12]	2018	DC 30 V and AC 15 V_P_	11	-
Ling [13]	2019	DC 20 V and AC 1 V_P_	-	-
This work	2022	DC 5 V and AC 0.05 V_P_	5738	4.2 mV

## Data Availability

Not applicable.

## Supplementary Materials

The following supporting information can be downloaded at: https://www.mdpi.com/article/10.3390/mi13060928/s1, Details for fabrication.
