# Supplementary material for: Wafer-Level Vacuum-Packaged Electric Field Microsensor: Structure Design, Theoretical Model, Microfabrication, and Characterization
Source: Micromachines (Basel). 2022 Jun 11;13(6):928. doi: 10.3390/mi13060928 (PMC9229140; doi:10.3390/mi13060928)
Supplement: Supplementary file 1 [file micromachines-13-00928-s001.zip › micromachines-1703694-supplementary.pdf]

Details for fabrication

(a) Spin spr220 photoresist on the surface of SOI device layer at 1500 rpm and then the spr220 photoresist is exposed in ABM lithography machine for 5S. The thickness of photoresist is 3.1μm. Deep reactive ion etching (DRIE) is utilized to etch the SOI device layer with the protection of patterned spr220 photoresist to form the sealing ring, the driving electrodes, the shielding beams and the working electrodes.

(b) Remove the oxide layer below the driving electrodes and the working electrodes by vapor HF.

(c) Cr/Au with the thickness of 30nm/200nm was deposited on the glass surface of the prepared GOS wafer to form the protective layer. Spin AZ5214 photoresist on the silicon layer of GOS wafer at 3000 rpm and then the AZ5214 photoresist is exposed in ABM lithography machine for 2S. The thickness of photoresist is 1μm. And then reactive-ion etching (RIE) with SF6 gas is used to form alignment marks on the silicon surface of the GOS wafer with the protection of patterned AZ5214 photoresist, which are used for the alignment of anodic bonding. The time of RIE is 6min and the etching depth of the mark is 2μm. Then clean the photoresist in both sides of the GOS.

(d) Spin AZ4620 photoresist on the glass layer of GOS wafer at 3000 rpm and then the AZ5214 photoresist is exposed in ABM lithography machine for 10S. The thickness of photoresist is 6μm. The Cr/Au deposited on the glass surface of the GOS is patterned using the metal corrosive fluid with the protection of the patterned AZ4620 photoresist to form the mask. And then the glass layer of the GOS is patterned utilizing vapor HF with the protection of the patterned Cr/Au mask. The etching depth in the glass layer of the GOS is 50μm.

(e) Ti/Au with the thickness of 1μm/30nm getter is deposited with the protection of a hard mark, which is patterned by a laser process.

(f) The anodic bonding of the SOI wafer and the GOS wafer in a vacuum environment is conducted and the Ti/Au getter is activated during the bonding process. The bonding voltage is 400V, the bonding temperature is 350℃, and the bonding pressure is 500mbar.

(g) Spin AZ4903 photoresist on the silicon layer of GOS wafer at 3000 rpm and then the AZ5214 photoresist is exposed in ABM lithography machine for 15S. The thickness of photoresist is 9.6μm. DRIE is utilized to etch completely through the GOS's silicon layer from the backside, and the etch stops at the surface of the GOS's glass layer. And then vapor HF is utilized to etch the exposed GOS's glass layer from the backside with the protection of the patterned GOS's silicon layer.

(h) Al Metal pads with the thickness of 1μm are deposited in the via holes with the protection of another hard mark and wire bonding is conducted.
